# Enhanced trends in spectral greening and climate anomalies across Europe

**DOI:** 10.1007/s10661-022-10853-8

**Published:** 2023-01-04

**Authors:** Michael Kempf

**Affiliations:** 1 Department of Geography, Physical Geography - Landscape Ecology and Geoinformation, University of Kiel, Kiel, Germany; 2 CRC1266-Scales of Transformation, Project A2 ‘Integrative Modelling of Socio-Environmental Dynamics’, University of Kiel, Kiel, Germany

**Keywords:** NDVI, Greening trend, Climate change, Drought, Landcover change, Climate extremes

## Abstract

Europe witnessed a strong increase in climate variability and enhanced climate-induced extreme events, such as hot drought periods, mega heat waves, and persistent flooding and flash floods. Intensified land degradation, land use, and landcover changes further amplified the pressure on the environmental system functionalities and fuelled climate change feedbacks. On the other hand, global satellite observations detected a positive spectral greening trend—most likely as a response to rising atmospheric CO_2_ concentrations and global warming. But which are the engines behind such shifts in surface reflectance patterns, vegetation response to global climate changes, or anomalies in the environmental control mechanisms? This article compares long-term environmental variables (1948–2021) to recent vegetation index data (Normalized Difference Vegetation Index (NDVI), 2001–2021) and presents regional trends in climate variability and vegetation response across Europe. Results show that positive trends in vegetation response, temperature, rainfall, and soil moisture are accompanied by a strong increase in climate anomalies over large parts of Europe. Vegetation dynamics are strongly coupled to increased temperature and enhanced soil moisture during winter and the early growing season in the northern latitudes. Simultaneously, temperature, precipitation, and soil moisture anomalies are strongly increasing. Such a strong amplification in climate variability across Europe further enhances the vulnerability of vegetation cover during extreme events.

## Introduction

Droughts are among the most severe environmental disasters, enhancing ecological and socio-economic conflicts (Geist & Lambin, 2004; Godfray et al., 2010; Gupta et al., 2020; Kaczan & Orgill-Meyer, 2020; Lambin & Geist, 2006; Lesk et al., 2016; Naumann et al., 2021; Schmidhuber & Tubiello, 2007; Xu et al., 2020). Particularly, long-term drought spells, hot drought events, flash droughts, and mega-heatwaves like in 2003, 2010, between 2018 and 2020, and recently in summer 2022 provoke persistent landcover transformations that amplify faunal and floral aggravation and land degradation, and consequently force socio-cultural adaptation (Barriopedro et al., 2011; Fischer et al., 2021; Ionita & Nagavciuc, 2021; Lin et al., 2020; Luterbacher et al., 2004; Miralles et al., 2014; Pereira et al., 2017; Quesada et al., 2012; Rasmijn et al., 2018; Schumacher et al., 2019; Shah et al., 2022; Sousa et al., 2020; Zhou et al., 2019). Parallel to this, the number of severe flood events and the frequency of wet spells increased over the past decades with significant regional variability across Europe (Breinl et al., 2020; Dai et al., 1998; Dietze et al., 2022; Kahle et al., 2022; Zolina et al., 2010). The development of rapidly occurring and long-lasting extremes poses particularly high challenges on the ecosystem’s adaptive mechanisms. Due to the accelerated speed, intensification, and persistence of dry and wet spell transitions, severe water shortages or massive oversupply occur within short-term periods (Shah et al., 2022). Recent results, for example, by Fischer et al. (2021), emphasized the strong increase in climate extremes probability and the short return periods of record-shattering events predicted for the next decades (Fischer et al., 2021).

Parallel to this, on the other hand, satellite observations and vegetation response monitoring detected enhanced global spectral greening and a generally positive trend in surface reflectance patterns, which shows that previously degraded landscapes were affected by anthropogenic signals superimposed on causative precipitation trends (Cortés et al., 2021; Forzieri et al., 2017; Herrmann et al., 2005; Kolecka, 2021; Myers-Smith et al., 2020; Piao et al., 2020). But vegetation response to global warming and rising atmospheric CO_2_ concentration is strongly coupled to regional climatic feedbacks and topographic variables (Forzieri et al., 2017; Myers-Smith et al., 2020; Piao et al., 2020). Anthropogenic forcing of spectral greening trends can regionally be linked to intensified land use, forest management (Chen et al., 2019; Correa-Díaz et al., 2021; de Jong et al., 2012; Piao et al., 2020), or even land abandonment (Kolecka, 2021). Particularly, crop production, which accounts for over one-third of the green-leaf area increase, highlights the spatial and seasonal variability of global greening trends (Chen et al., 2019; Winkler et al., 2021). Extensive monoculture crop cultivation increases the yield vulnerability to climate extremes and persistent heat waves, which in turn can cause massive harvest loss (Brunner et al., 2018; Gampe et al., 2021).

Most of these system control mechanisms function on the very regional scale but both surface reflectance and environmental parameter trends show similar behaviour across Europe. That differs substantially from the strong increase in climate anomalies that have been recorded over the past decades. Long-term supraregional climate trends most likely obfuscate regional vegetation response anomalies during persistent drought periods and underestimate local environmental feedbacks. There is a need to monitor both time series of climatic parameters and multiannual variability and anomalies to estimate the increasing vulnerability of surface cover to climate change. This paper evaluates long-term spectral greening as well as environmental trends and anomalies across Europe using long-term monthly vegetation indices (Normalized Difference Vegetation Index, NDVI) and monthly climate and environmental variables (Global Land Data Assimilation System, GLDAS) (Beaudoing et al. 2020; Harris et al., 2020; Kumar et al., 2006; Peters-Lidard et al., 2007; Rodell et al., 2004) (Table 1).

**Table 1:** GLDAS (Global Land Data Assimilation System, Version 2) variables and units (Beaudoing et al. 2020; Kumar et al., 2006; Peters-Lidard et al., 2007; Rodell et al., 2004)

	Variable ID	Standard name	Long name	Unit	
GLDAS variable	Snowf_tavg	snowfall_flux	Snow precipitation rate	kg/m^2^/s	Climate data interface,version 1.9.8, GLDAS_NOAH025_M, land surface model, L4 monthly 0.25 × 0.25 degree V2.1
	Rainf_tavg	rainfall_flux	Rain precipitation rate	kg/m^2^/s	
	AvgSurfT_inst	surface_temperature	Average Surface Skin temperature	kg/m^2^/s	
	SnowDepth_inst	surface_snow_thickness	Snow depth	m	
	SoilMoi0_10cm_inst	soil_moisture_content	Soil moisture	kg/m^2^	
	SoilMoi10_40cm_inst	soil_moisture_content	Soil moisture	kg/m^2^	
	SoilTMP0_10cm_inst	soil_temperature	Soil temperature	kg/m^2^	
	Tair_f_inst	air_temperature	Temperature	kg/m^2^	

## Material and methods

Monitoring vegetation change through remote sensing techniques has become a common tool since the introduction of the NDVI and the availability of medium-resolution satellite imagery covering the past two decades (Cortés et al., 2021; Tucker, 1979; Tucker et al., 1986). For this article, monthly MODIS (Moderate Resolution Imaging Spectroradiometer) NDVI imagery was analysed to detect vegetation anomalies compared to background spectral greening and browning trends and precipitation and temperature variability as well as environmental covariates over the period 2001–2021.

Spatial and temporal subsets of NDVI data (2001–2021) and environmental variables from GLDAS (1948–2021) (Beaudoing et al. 2020) were calculated. Annual and seasonal trends as well as anomalies were processed for each environmental subset with growing seasons as March–May (MAM), June–August (JJA), September–November (SON), and December–February (DJF, including January and February 2022). The NDVI subsets were analysed for surface reflectance anomalies and spectral greening and browning trends based on the MODIS NDVI time series with 252 single images. In general, the NDVI is an index to distinguish the photosynthetic activity of vegetation from other land surfaces and acts as a vegetation performance indicator where higher values indicate higher photosynthetic activity (Justice et al., 1985; Tucker, 1979). The index is based on the reflection characteristics of near-infrared (NIR) and the absorption of red radiation (Red) (Anyamba & Tucker, 2005; Tucker, 1979; Tucker et al., 1991). All statistical analyses were performed using the R environment (R: a language and environment for statistical computing. R Foundation for Statistical Computing, Vienna, Austria. URL https://www.R-project.org/). All codes supporting the analyses are stored in a repository (Kempf, 2022). The data underlying the analysis are freely available on the Internet.

### MODIS NDVI monthly dataset

Global NDVI monthly time series datasets (2001–2021) were downloaded from the Earthdata server of the United States Geological Survey (USGS) (https://lpdaac.usgs.gov/products/mod13c2v006/, last accessed 3rd of July 2022) (Didan 2015). The NDVI data consist of cloud-free composites with 0.05° geographic grid resolution (5600 m) on a monthly return period. Annual composites and growing season subsets were calculated from the data.

### GLDAS

GLDAS climate and environmental variable datasets were acquired from the Goddard Earth Sciences Data and Information Services Center (GLDAS, Noah Land Surface Model L4 monthly 0.25 × 0.25 degree V2.1 (GLDAS_NOAH025_M, https://disc.gsfc.nasa.gov; last accessed 3rd of July 2022) (Beaudoing et al. 2020; Rodell et al., 2004). The data covers the period 1948–2021 and the whole temporal range was used to visualize trends and anomalies over the past 70 years in Europe. Each environmental subset was extracted and annual composites and growing season means were calculated accordingly (see Table 1).

### Trend analysis

Trend analyses in surface reflection patterns and environmental explanatory covariates were performed using a linear model and annual and seasonal composite raster stacks (Brandt et al., 2014). Monthly data was aggregated to produce annual and seasonal means, and the slope was calculated. *P* values were extracted and all values *p* > 0.05 were masked to show trends with a 95% confidence level. This produces a raster with positive or negative trends with temporal reference to the entire observation period (1948–2021, GLDAS, and 2001–2021, NDVI). To visualize the temporal development over Europe, monthly mean values were calculated and plotted as annual and seasonal time series. For this reason, two spatial masks were created that distinguish into regions below and above the mean annual average temperature of the study area over the period 1948–2021 (7.046578 °C). Basically, this is a differentiation into a northern and a southern part but includes higher elevated areas into the lower annual average temperate range.

### Anomaly analyses

To produce annual and seasonal anomalies, the arithmetic mean (*m*) and the first standard deviation (*SD*) were calculated from aggregated raster stacks of each variable. *SD* was subtracted from and added to the mean value (*m − SD*; *m* + *SD*) to create the range of the standard deviation for the reference period. The (*m* + *SD*) was subtracted from each single year and growing period value and all values ≤ 0 were removed for the upper limits of the standard deviation range. According to the lower limits of the standard deviation range, (*m − SD*) was subtracted from each year and growing period value and all values ≥ 0 were removed. Both parts were merged to show negative and positive trends. Eventually, raster mean values of negative and positive anomalies were extracted and visualized with a smoothing estimator of the *ggplot2* package that uses a linear relationship between x and y (LOESS) (Wickham, 2009).

### Correlation

The NDVI annual sums were correlated with the selected GLDAS variables using a pixel-wise Spearman rank-based correlation. For this reason, the NDVI raster stacks were resampled to 0.5° × 0.5° to fit the GLDAS spatial resolution. A correlation was performed to detect interdependency between surface reflectance and environmental control mechanisms. Similar approaches have recently been reported, for example, from Bavaria (Kloos et al., 2021) and Central Asia (Peng et al., 2021; Shen et al., 2021).

## Results

Surface reflectance and vegetation greening trends and trends in environmental controlling parameters are inherently interconnected, e.g. through soil moisture deficits or precipitation and temperature development (Dari et al., 2021; Lian et al., 2020). In the following, the results of the NDVI and environmental trend analysis are presented in spatial and temporal resolution, covering the periods 2001–2021 and 1948–2021 respectively. The correlation of all input variables with NDVI time series data is presented followed by annual and seasonal anomalies.

### Surface reflectance and environmental trends in Europe

**Fig. 1:**
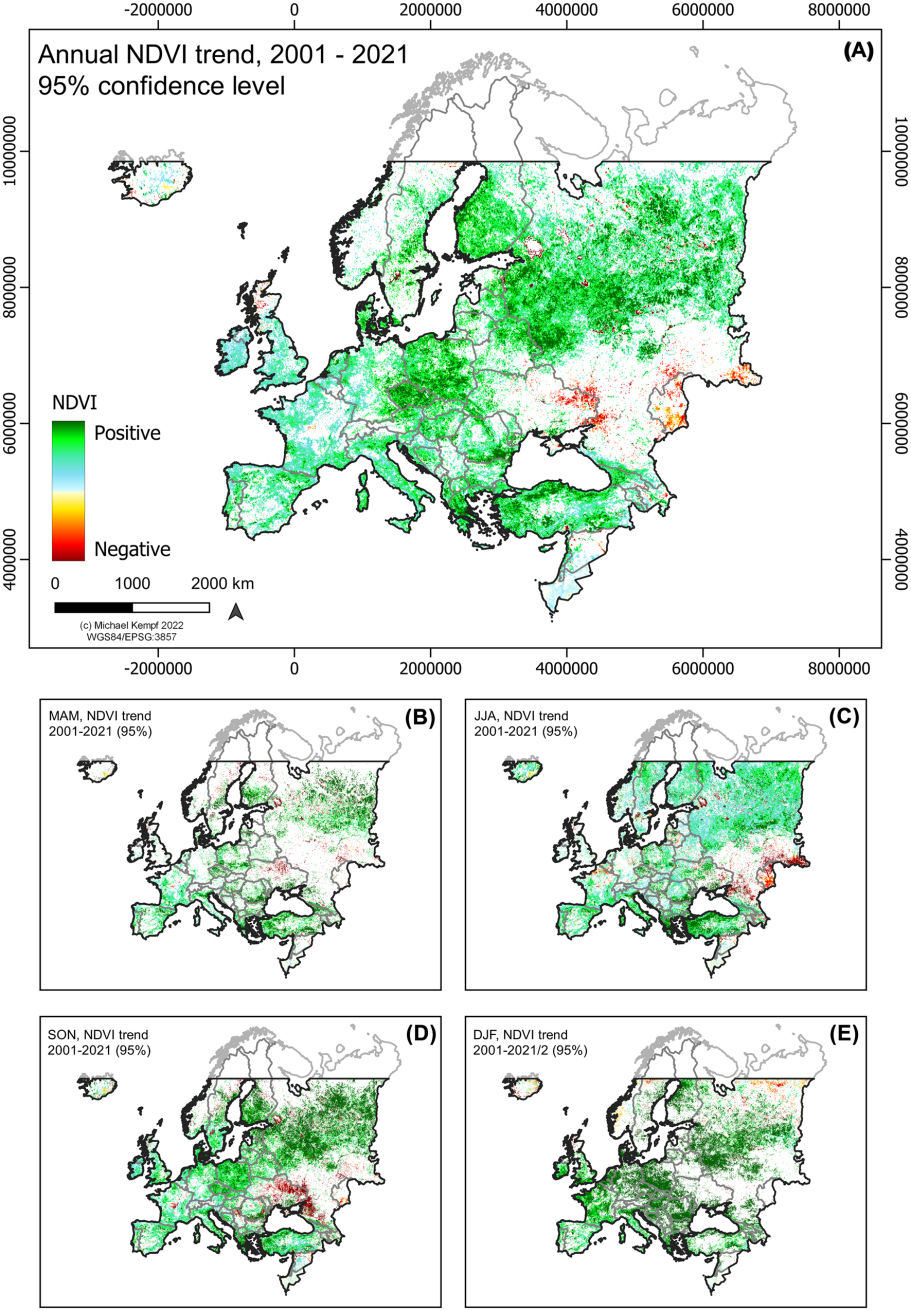
**A** Spectral surface reflectance trends derived from annual NDVI composites (95% confidence level) across Europe for the period 2001–2021/22. **B**–**E** Seasonal NDVI trends, March–May (MAM), June–August (JJA), September–November (SON), and December–February (including January and February 2022) (95% confidence level)

Climate change is affecting vegetation growth behaviour depending on the geographical location with generally positive trends in Northern Europe and negative trends in Southern Europe. Particularly, Northern Europe experiences a trend towards longer growing seasons, triggered by increasing temperatures, longer summer duration, and rising CO_2_ concentrations (Hyvönen et al., 2007; Venäläinen et al., 2022). Southern Europe, however, is expected to increasing dry-up processes and enhancing wildfire and erosion vulnerability, which accounts for severe and large-scale ecosystem disturbances (Górriz-Mifsud et al., 2022). Spectral surface reflectance patterns derived from NDVI trend analysis show strongly positive development in most parts of continental Europe during the period 2001–2021 (Fig. 1). Particularly strong trends can be detected in the central and north- and southeastern parts. Western Europe and large parts of France, the Iberian Peninsula, Italy, and the British Islands show weaker but still significant spectral greening trends. Eastern Europe shows a strongly negative surface cover response and an increasing spectral browning trend with significant peaks in southeastern Ukraine and Russia. The strongest greening trends occur during SON in Northeastern Europe and during DJF in large parts of Central Europe. Conversely, the very eastern part of continental Europe experiences strongly negative surface reflection trends between the onset of the growing period in March until the end of November (Fig. 1B, C).

**Fig. 2:**
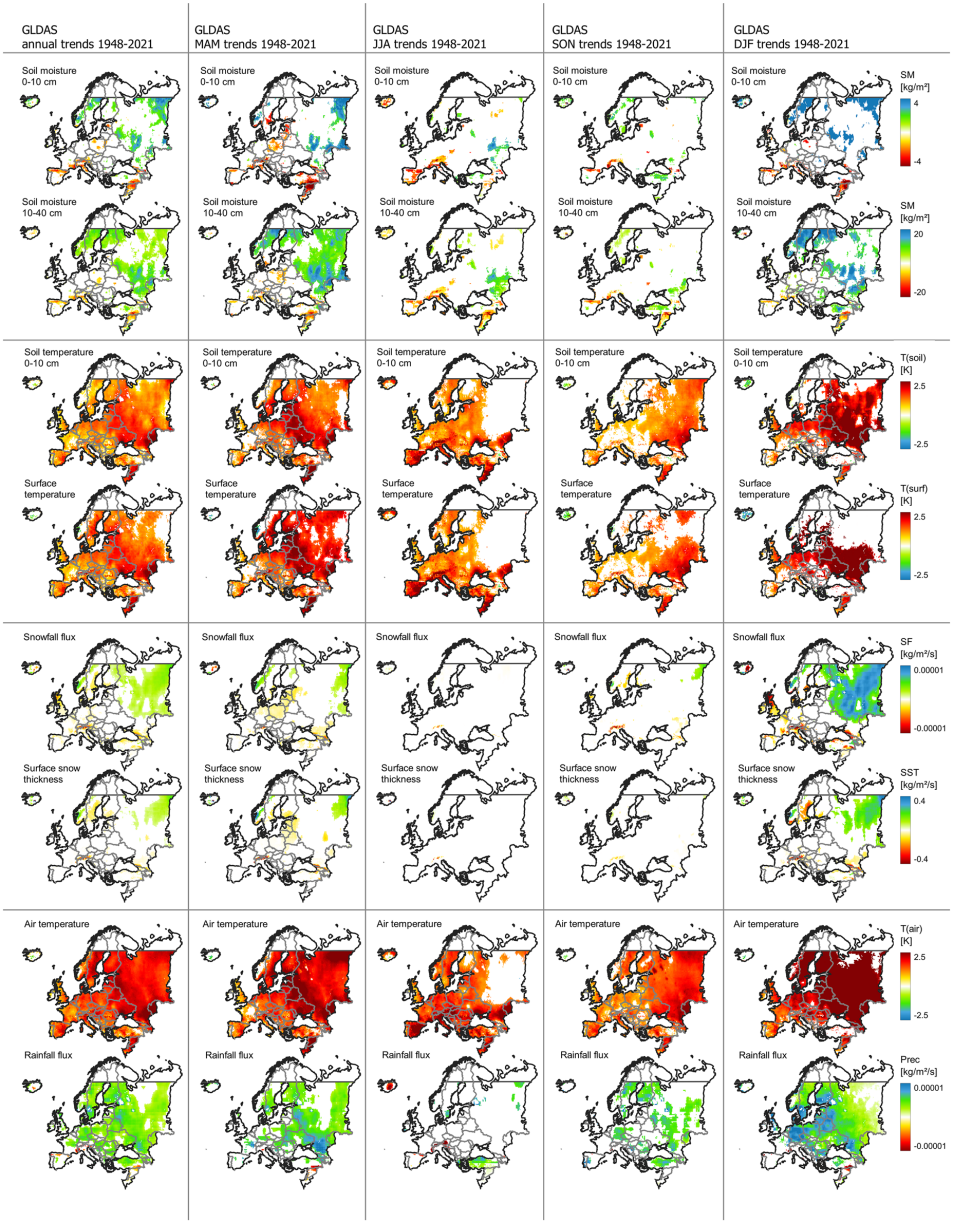
Annual and seasonal trend analysis of selected GLDAS variables across Europe (1948–2021). MAM, March–May; JJA, June–August; SON, September–November; DJF, December–February (including January and February 2022). Visualized trends are 95% confidence level. From top: soil moisture (0–10 cm), soil moisture (10–40 cm), soil temperature (0–10 cm), surface temperature, snowfall flux (snowfall precipitation rate), surface snow thickness, air temperature, and rainfall flux (rainfall precipitation rate)

**Fig. 3:**
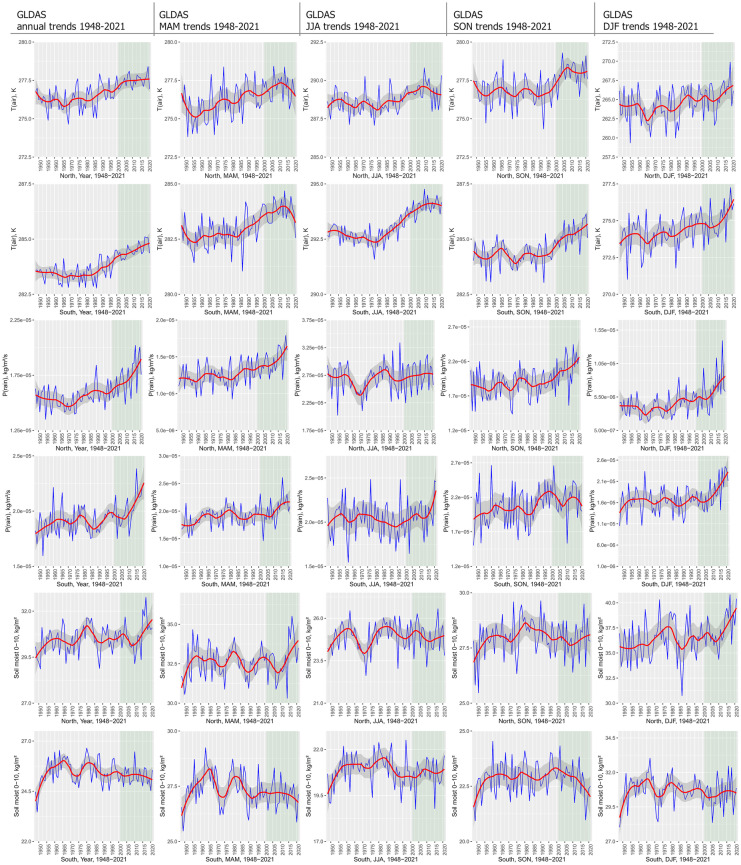

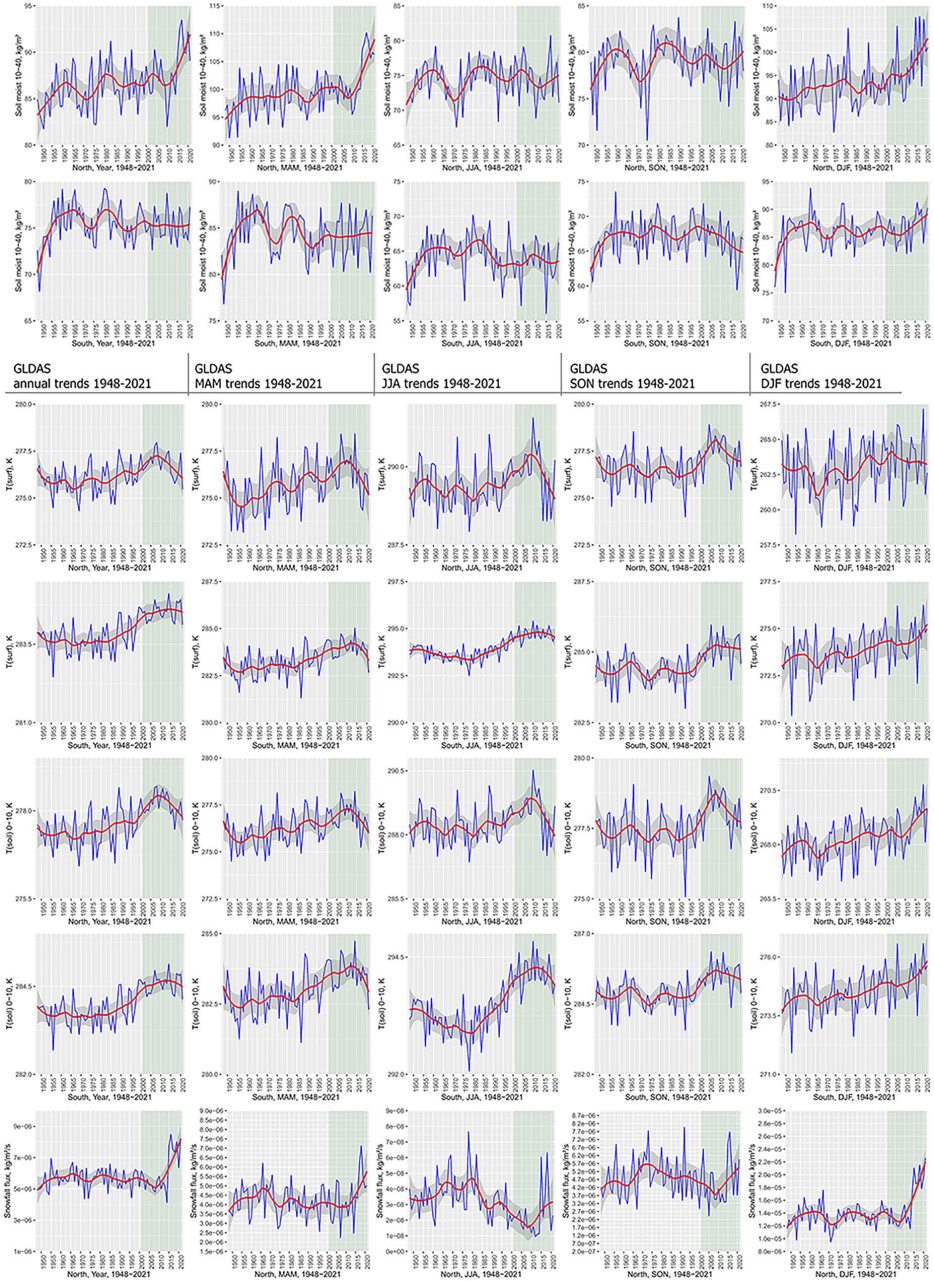

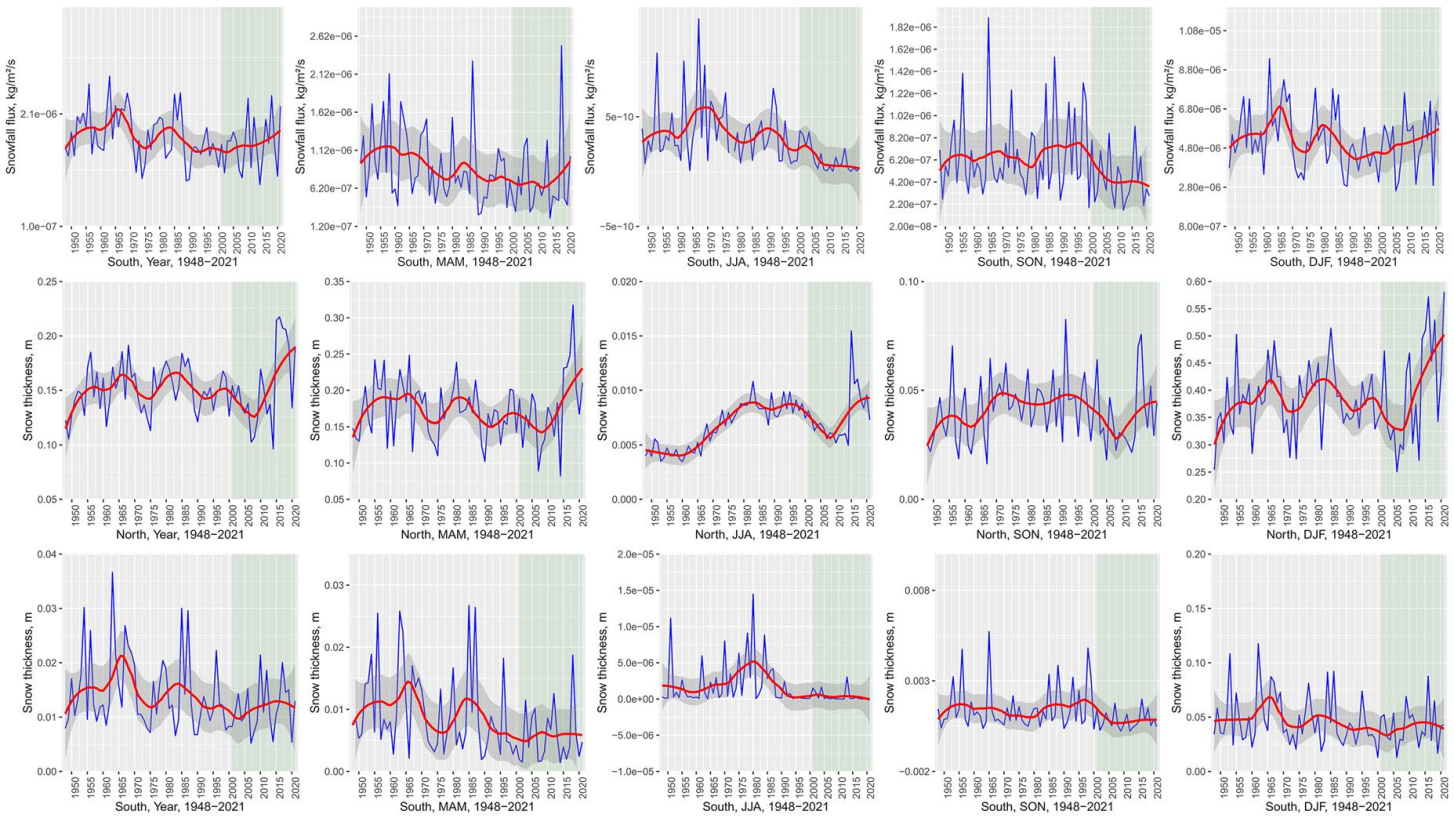
**a** GLDAS variable annual and seasonal trend mean values in Europe over the period 1948–2021 (DJF including January and February 2022). The data was analysed using spatial masks of temperature thresholds (study area average temperature). *Y* axis breaks vary according to seasonal variability. The NDVI reference period 2001–2021 emphasized in light green. From top: air temperature, rainfall flux (rainfall precipitation rate), soil moisture (0–10 cm), and soil moisture (10–40 cm). **b** GLDAS variable annual and seasonal trend mean values in Europe over the period 1948–2021 (DJF including January and February 2022). The data was analysed using spatial masks of temperature thresholds (study area average temperature). *Y* axis breaks vary according to seasonal variability. The NDVI reference period 2001–2021 emphasized in light green. From top: surface temperature, soil temperature (0–10 cm), snowfall flux (snowfall precipitation rate), and surface snow thickness

Significant warming trends can be detected in most parts of continental Europe and particularly in the eastern and northeastern parts (Fig. 2). Temporal development further emphasizes continuous temperature increase at least since the 1970s with a recent acceleration starting before 2000 (Fig. 3a, b). All three temperature variables (T(air), T(surf), and T(soil)) included in the analyses show a strong increase in annual trends with T(air) exceeding T(surf) and T(soil). Interannual variability is largest during DJF and MAM with a particular temperature increase during the winter months, affecting particularly the eastern and northern parts of the continent (Venäläinen et al., 2022). During the summer months (JJA), Southern Europe and particularly the Mediterranean are affected by a strong increase in T(air) and T(surf) (Vogel et al., 2021). This becomes evident from the strong temperature increase displayed by the masked southern temperature trends (Fig. 3a). This trend is less pronounced in the northern part of the study area. Summer extreme temperatures in Southern Europe are inherently linked to soil moisture content (Materia et al., 2022). Both 0–10 and 10–40 cm depth soil moisture show negative trends in Southern Europe and the Mediterranean during JJA—compared to the positive continental annual mean trend. Particularly striking are the differences in MAM soil moisture trends between southern and Northern Europe. While Northern Europe experiences an increase in early growing season soil moisture, probably related to increasing rainfall and early snow melt, southern MAM soil moisture content decreases. Both snowfall flux and rainfall precipitation are significantly positive during MAM, which contributed to the positive soil moisture trend (Fig. 3b). According to Lian et al. (2020), soil moisture deficits in summer can be traced back to intensified crop production and foliage cover over the previous months, which would account for the positive greening trends probably linked to irrigation measures (Cramer et al., 2018; Pool et al., 2021). The effects of irrigation, climate change, decreased soil moisture, and precipitation totals have further been reported from special crops, such as truffles (Büntgen et al., 2019).

These southern summer drying-up trends are contrasted by the intensification of soil moisture especially during the early growing season and the winter months in large parts of Eastern Europe. However, precipitation increase is assumed to be compensated by increasing evapotranspiration, particularly enhancing summer drought risk (Venäläinen et al., 2022). Synchronously, a strong increase in snowfall flux (snow precipitation rate) and a general increase in rainfall (rain precipitation rate) can be observed during MAM and DJF, affecting both the eastern European countries and large parts of Central Europe. The positive Central European precipitation trends during DJF, however, do not cause a significant increase in soil moisture (Fig. 3a). Most likely, soil moisture development is controlled by enhanced winter snowfall and increased surface snow thickness. In combination with higher temperatures and rising humidity transport, this contributes to accelerated plant growth and phenological shifts in Eastern Europe during summer. Positive NDVI trends would therefore not necessarily indicate a significant decrease in snow cover during winter but rather a stronger vegetation response to increased warming and soil moisture content, advanced onset of the early phenological phase, and thawing processes of the permafrost (Fonti et al., 2021; Rosbakh et al., 2021).

Annual rainfall variability shows positive trends over Central Europe and the eastern and Northern European countries (Fig. 2). Southern France, the Alps, northern Spain, and the Levant show significantly negative precipitation trends. Strong and significant increase in total precipitation (rainfall rate) occurs during MAM and DJF with the strongest increase during winter at least since 2010 (Fig. 3a). The summer months, however, show a significant decrease in parts of the Mediterranean and the Alpine region.

### Correlation and attribution of climatic variables to spectral greening

Spearman’s rank-based correlation was performed between the environmental variables and the NDVI time series over the period 2001–2021. Due to the limited available sample size of the NDVI time series, the correlation values can only provide a rough indication of cause and effect between the variables. However, the results from the trend analyses can be used to strengthen the reliability of their physiological connection. The trend analyses have emphasized the strong acceleration of environmental change after 2000, which is then visible in the vegetation response across Europe.

**Fig. 4:**
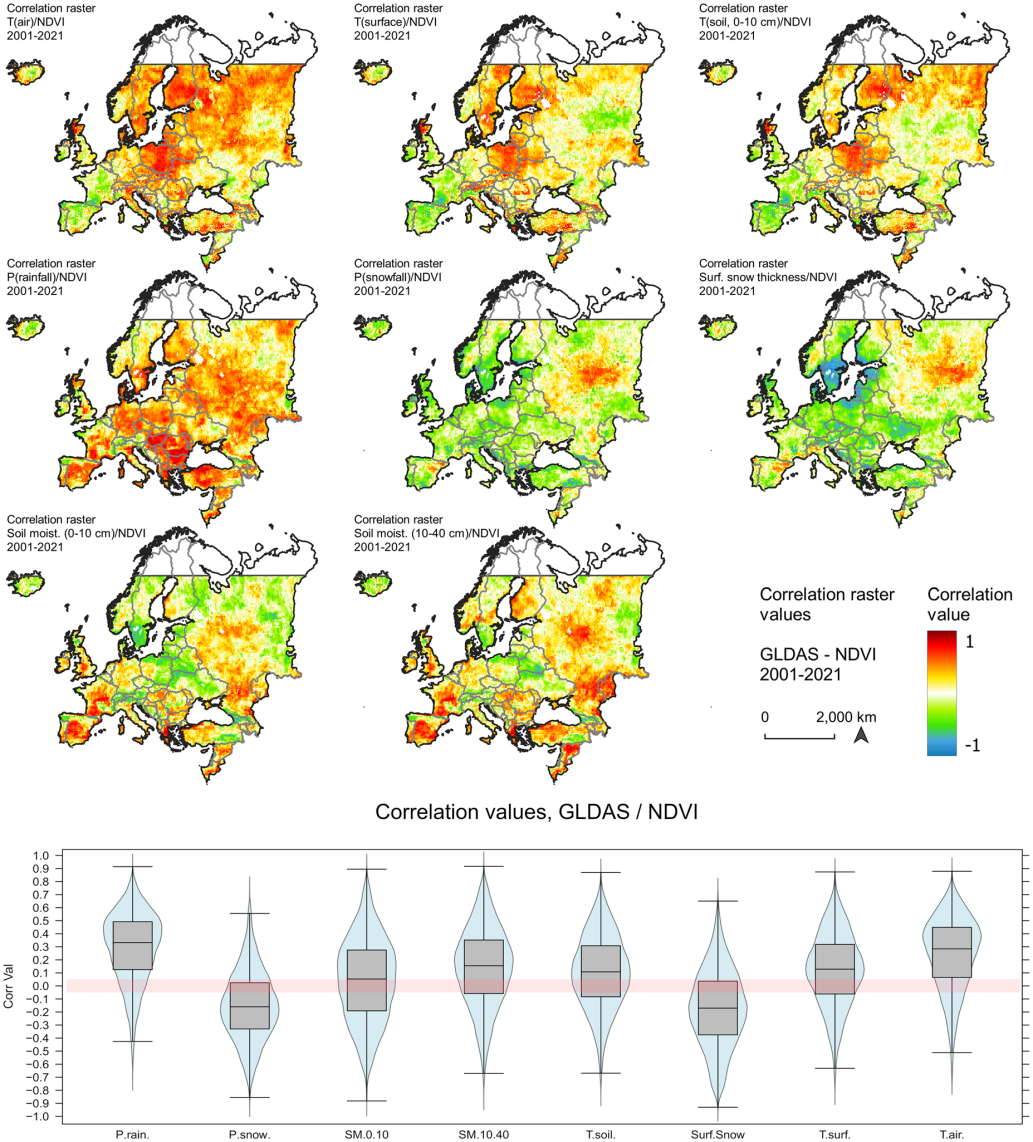
Correlation values between NDVI and GLDAS variables for the period 2001–2021 in Europe

The severe increase in temperature in the Mediterranean (e.g. JJA T(air)) is not strongly correlated with spectral greening (Fig. 4). However, there is a strong correlation between rainfall and soil moisture and NDVI signal, which shows that vegetation response in Southern Europe and large parts of France is mostly controlled by soil moisture content. Soil moisture content is furthermore the limiting vegetation growth factor in Ukraine and continental Eastern Europe that experiences a very strong decline in surface spectral greening trends over the past two decades. Recent results, however, have emphasized the strong effect of illegal vegetation burning prior and after crop cultivation in Ukraine, which could enhance the decline of spectral greening signals (Hall et al., 2021).

Across the eastern European countries and Russia, NDVI is positively correlated with snowfall and surface snow cover thickness, which in turn contributes to soil moisture content during the early growing season (MAM). In Central and Northeastern Europe, a temperature-controlled vegetation response signal is visible from the analyses. Particularly, Poland, Scandinavia, and large parts of Russia are affected by a strong increase in JJA and DJF temperature. The strongly positive NDVI signals in Central Europe can most likely be linked to reduced snowfall and snow cover in winter and particularly during early spring, which enhances the surface reflectance. A shift in the phenological phases, an advanced onset of the growing season, and intensive irrigation during JJA can further influence the NDVI signal, giving the impression of intensified spectral greening. Similarly to the Mediterranean region, Central Europe experiences a drop in soil moisture, which could probably be explained by enhanced irrigation, crop production (e.g. potatoes and maize), and foliage cover (Yu et al., 2020; Zajac et al., 2022).

### European climate and environmental variability

**Fig. 5:**
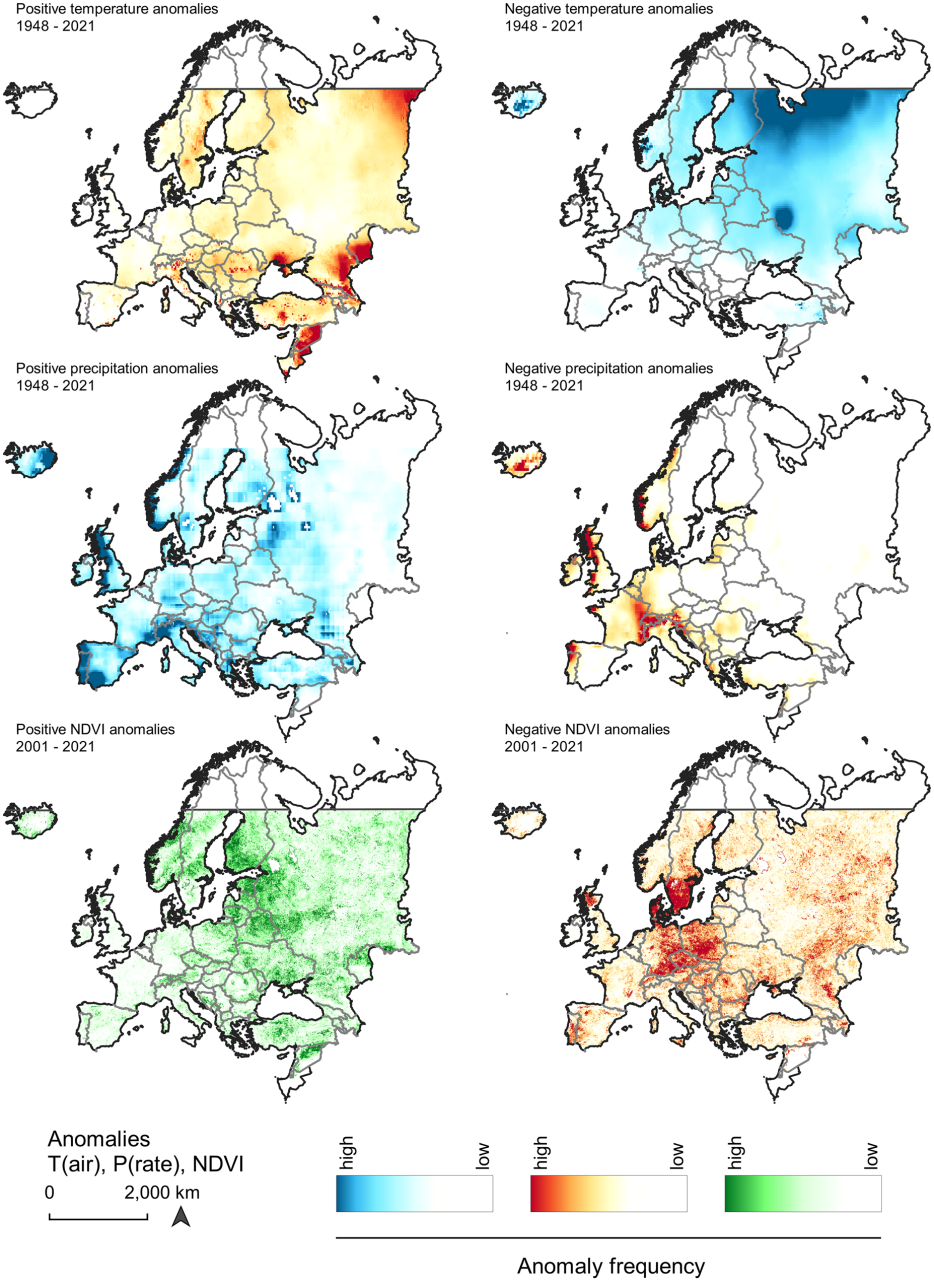
Temperature (T(air)), precipitation (P(rate)), and NDVI anomaly frequency between 1948–2021 and 2001–2021 across Europe

**Fig. 6:**
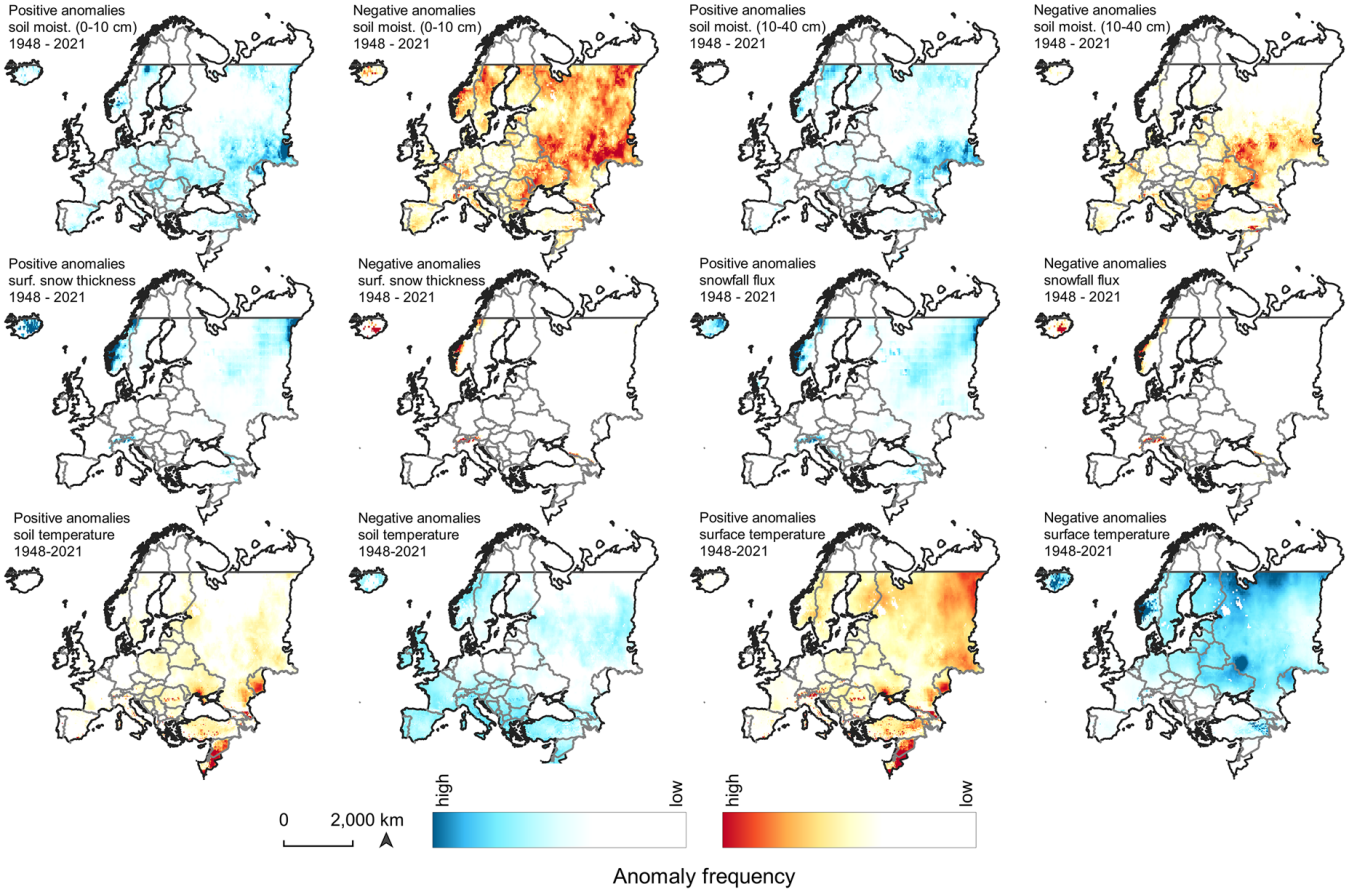
Positive and negative anomaly occurrences of selected GLDAS environmental variable across Europe (1948–2021)

Temperature anomalies (T(air)) increase across Europe, affecting both the southern as well as the northern regions, and subsequent hot and dry year periods are estimated to occur more frequently by the mid of the twenty-first century (Gampe et al., 2021; Vogel et al., 2021). Positive anomalies occur frequently across most parts of Europe and particularly in the Mediterranean, the Balkan, Turkey, the Levant, Ukraine, southern and northern Russia, and strongly in the eastern parts of continental Europe—affecting tree growth and enhancing forest dieback (Del Martinez Castillo et al. 2022) (Fig. 5). A strong local increase of T(air) anomalies can be observed in Sweden, stretching from southern Scandinavia across middle Sweden towards the north. Simultaneously, positive T(surf) anomalies are occurring across the entire Scandinavia. In Central Europe, it is particularly northern Italy and regions towards Romania which are affected by high-temperature anomalies. Soil moisture shows the strongest negative anomalies across Russia, Ukraine, and towards the Black Sea, affecting more the upper soil layer (0–10 cm) (Fig. 6). In the west, Scandinavia is more negatively impacted by increased soil moisture variability. Negative temperature anomalies are much smoother distributed across Europe with a gradual increase towards the northeastern parts of the continent. There is, however, a significant cold-spot anomaly located at the border between Russia and Ukraine. An increase in temperature variability strengthens the risk of crop failure during periods of either hot droughts or intensified cold waves (Piticar et al., 2018; Vogel et al., 2019).

Parallel to this, rainfall anomalies (P(rain)) are increasingly frequent in coastal areas of the Iberian Peninsula, the UK, and the southern Alpine region stretching south across the Balkans. However, dry spells and negative P(rain) anomalies occur in the same regions, which emphasizes the very strong climate variability in large parts of Central Europe. An increase in winter heavy precipitation has been predicted in Western Europe for future climate models, particularly affecting the UK and parts of southwestern Scandinavia (Chan et al., 2018; Christidis et al., 2021; Ketzler et al., 2021; Whan et al., 2020). Despite the changes in mean precipitation trends, the annual variability strongly increases (Chan et al., 2018; Zhang et al., 2021).

Positive surface reflectance anomalies can be detected across Central and large parts of Eastern Europe with a maximum in the Baltic Countries and southern Finland. Strongly negative anomalies occur mostly in southern Sweden and Denmark, and Central Europe. Particularly, southern and eastern Germany, Czech Republic, and Poland are affected by negative vegetation cover anomalies over the period 2001–2021. At the same time, snowfall flux anomalies (P(snow)) are increasingly positive across Russia and Norway. The Alpine region, however, shows a significantly negative tendency to snowfall and snow cover anomalies, according to the trend analysis.

**Fig. 7:**
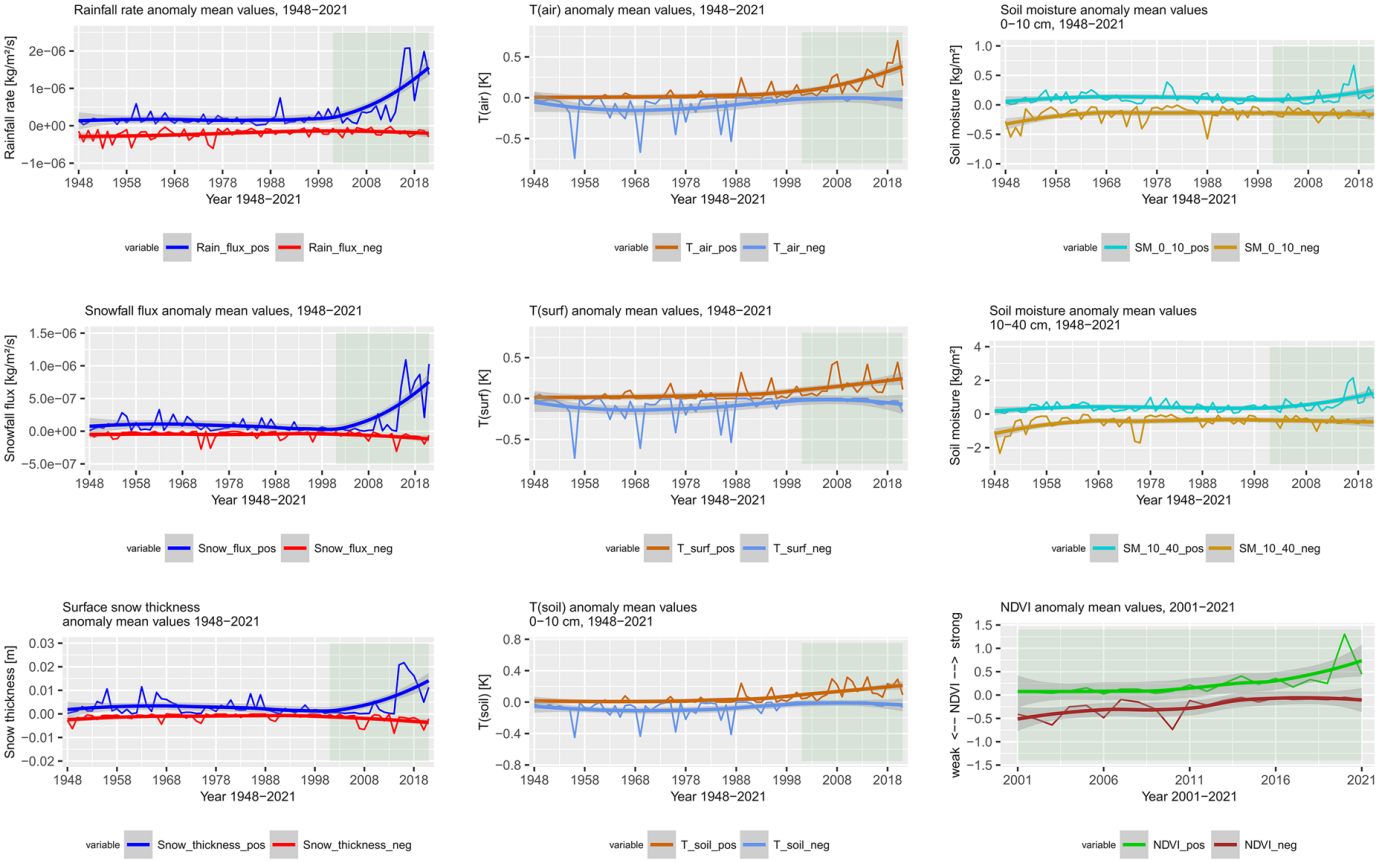
Time series of GLDAS (1948–2021) and NDVI (2001–2021) anomaly means across Europe. Both negative and positive anomaly mean values are plotted and smoothed with a local smoothing estimator (LOESS)

Long-term time series analysis of the selected GLDAS variables over the period 1948–2021 shows a strong increase in positive rainfall and snowfall flux (P(rain), P(snow)), followed by an increase in positive snow cover thickness (Fig. 7). Mean negative precipitation rate anomalies are slightly decreasing. The massive increase in positive anomalies is most evident after 2000, indicating a strong increase in wet spells and precipitation extreme events across Europe. Parallel to this, T(air), T(surf), and T(soil) show enhanced positive anomalies over the same period, pointing towards increased dry spells and hot drought events. Negative temperature events are almost lacking in the recent continental mean anomaly time series—compared to events prior to 1990. Soil moisture shows increased variability, particularly in recent years. Accordingly, NDVI values fluctuate strongly, with increasingly positive frequency and no increase in negative occurrences.

## Discussion

Remote sensing tools have gained momentum in large-scale observations of vegetation response to global warming, greening trend analyses, and landcover change monitoring. The recent trends in spectral greening and browning have been linked for example to surface water body fraction, increased temperature, atmospheric carbon input, and soil moisture content (Forzieri et al., 2017; Li et al., 2021). However, satellite-based remote sensing observations mostly build on coarse-grained NDVI pixel datasets, which are mixed data composites that can only provide an estimate of the actual land cover, particularly in climate-sensitive areas such as shrubland or tundra (Herrmann & Tappan, 2013; Nelson et al., 2022). But open-source NDVI time series data are highly suitable for supraregional monitoring of spectral surface reflection variability and allow the linkage to environmental and climate driving factors, such as soil moisture content and water availability (Chen et al., 2019; Kloos et al., 2021; Li et al., 2021; Peng et al., 2021)—important factors that amplify climate-induced extreme weather events.

### Climate change and ecosystem feedbacks across Europe

The discussion about the cause and effect of the local and regional anthropogenic overprint on the ecosystem’s functionalities, fuelled by global climate change feedbacks, has been reinforced by the strong gradient of continental European flood and drought spells (Ciais et al., 2005; Harris, 2010; Herrmann & Hutchinson, 2005; Lin et al., 2020; Zahradníček et al., 2015). Particularly, the severe drought episodes in 2003, 2010, between 2018 and 2020 (and this year, 2022), and followed by dramatic flooding events across Europe in summer 2021 have entered the political and economic debate (Brun et al., 2020; Büntgen et al., 2021; Cramer et al., 2018; Dirmeyer et al., 2021; Hanewinkel et al., 2022; Hari et al., 2020; Kahle et al., 2022; Kempf & Glaser, 2020). Temperature is constantly increasing across Europe and particularly over terrestrial surfaces. According to the 2021 IPCC report, the year 2020 has been marked as the warmest on instrumental record (Masson-Delmotte et al. 2021).

High temperatures paired with increasing soil moisture prolong the growing season in the high latitudes, which in turn amplifies the risk for windfall during storm season, bark beetle infections, and forest decline (Müller et al., 2008; Schelhaas et al., 2003; Venäläinen et al., 2022). Regional vegetation declined as a response to local climate change feedbacks, persistent drought periods, windthrow damage, and the subsequent impact of the massive spread of, for example, *Ips typographus L.* (Anderegg et al., 2013; Hlásny et al., 2021a, b). Particularly, predominant Norway spruce stands (*Picea abies* L. Karst) are prone to beetle outbreaks, due to their high vulnerability to drought periods, rising temperature, and increased storm occurrences under a globally changing climate of the boreal zones (Čada et al., 2016; Macek et al., 2017; Netherer et al., 2021). Extensive and monodominant spruce plantations further amplified the risk of severe outbreaks and large-scale tree mortality during periods of extreme temperature variability (Macek et al., 2017). Canopy dieback, on the other hand, opens up gaps in the forest, which increases light penetration and soil moisture due to decreased transpiration. Rising day-time temperatures further enhance the decomposition of organic matter, thus speeding-up forest regeneration and increasing species diversity (Muscolo et al., 2014; Przepióra et al., 2020; Scharenbroch & Bockheim, 2008).

Extreme heatwaves as in 2003 in Central Europe and particularly in 2010 across Russia have been traced back to atmospheric patterns that caused a massive increase in surface temperature (Di Capua et al., 2021; Liu et al., 2020). In Russia, constant warming over an increasingly desiccated land surface caused persistent thick hot-air masses that maintained surface warming and led to further heat accumulation and soil desiccation, amplifying the heatwave cascading effects (Christian et al., 2020; Miralles et al., 2014). Particularly, the southern parts, which experience a significant increase in heat supply and soil temperature and a strong reduction in soil moisture, are currently the most productive and intensely exploited wheat cultivation areas (Di Paola et al., 2018). Considering the trend towards increased soil moisture availability and temperature across the northern and northeastern parts of Russia, for example, land use patterns are most likely to shift in the future. This can cause strong surface transformation and a reverse trend in soil moisture availability under rapidly increasing surface and soil temperature.

### Anthropogenic impact on regional vegetation and surface change

Global warming and coupled rising CO_2_ atmospheric concentrations enhance ecosystem productivity in the high latitudes, increase the decomposition of organic matter in the permafrost region, and shift the climatic limitations of arable land towards the north (Liu et al., 2021). These feedbacks not only amplify CO_2_ and methane release from thawing permafrost (Knoblauch et al., 2021; Zhu et al., 2019), but also increase the carbon sink rate due to accelerated vegetation productivity during prolonged growing seasons in the forested zones of Northern Europe. Theoretically, this leads to the expansion of the boreal zone as well as the migration of temperate forest species into the boreal zone across the Northern Hemisphere (Boisvert‐Marsh and Blois 2021). However, the corridor of potentially arable land spreads synchronously, which opens up large-scale areas for crop cultivation that were previously covered with forest (King et al., 2018). Simultaneously, semi-arid regions become drier and land degradation, e.g. in the Mediterranean, is continuously increasing. Eventually, this triggers the release of CO_2_ and other greenhouse gases from bare soil surfaces and reduces the carbon sink during vegetation cover decline (Lal et al., 2021). While these changes are significant on the large scale across Europe, there are regional differences in NDVI trends that most likely get fuelled by regional land use strategies, land abandonment, or deforestation activity as coupled to human behaviour. In Poland, for example, recent results by Kolecka (2021) showed intensified greening trends for abandoned land compared to managed cropland. Here, land abandonment is not only caused by agriculturally low suitability but is rather more likely the result of unproductive farming techniques and economically challenging parcel size and shape from long-standing agricultural tradition (Czesak et al., 2021). Socio-economic development and political decision-making are particularly effective drivers of afforestation and land abandonment in the former Soviet Union (Ershov et al., 2022; Rolinski et al., 2021), which in turn have contributed to the regional carbon sink on the short-term scale. However, carbon release during re-opening for agricultural purposes (Schierhorn et al., 2013), forced by Russia’s political agenda in 2022 and probably continued in 2023 and beyond, can contribute significantly to the global carbon emissions.

### Future greening trends under climate change

European forests are highly vulnerable to rapid changes in ecosystem parameters caused by climate change (Forzieri et al., 2021). The observed response in plant growth and a shift towards arid-tolerant species does not necessarily mean a recovery in total ecological diversity (Herrmann & Tappan, 2013; Ibrahim et al., 2018; Zida et al., 2020). Partly, this trend is attributed to increased atmospheric CO_2_ concentration, precipitation totals, the spread of agricultural exploitation, and increased warming of the permafrost in the Northern Hemisphere (Peng et al., 2020; Piao et al., 2020; Winkler et al., 2021). Global spectral greening trends and phenological shifts over the past two decades were thought to emerge from increased greenhouse gas concentrations and higher temperatures, causing higher biological activity and intensified carbon sink in higher latitudes in the Northern Hemisphere (de Jong et al., 2012; Menzel et al., 2020; Piao et al., 2019; Rosbakh et al., 2021; Zhou et al., 2001). Results from high-resolution satellite LAI (Leaf Area Index) analyses identified forest regrowth following land abandonment as the main driving factor of EU-wide spectral greening (Buitenwerf et al., 2018). On the other hand, grassland and shrubs were particularly affected by an inverse trend, and according to de Jong and colleagues (2012), semi-arid regions are more vulnerable to browning trends after rapid greening caused by short-term climate variability (de Jong et al., 2012). In combination with persistent hot drought periods, the carbon uptake can be drastically reduced by decreased gross primary production (GPP), particularly affecting grassland and cropland in the northern temperate climate zone (Gampe et al., 2021). General grassland and cropland response to CO_2_ increase is particularly sensitive to soil moisture availability, soil type, and texture (Fay et al., 2012) and an increasing soil moisture trend across Northeastern Europe is linked to greening signals. Climate variability and particularly the precipitation rate after the growing season of the preceding year until August of the current year control productivity patterns (Li et al., 2013). Recent results have further narrowed down the temporal corridor of European species’ phenology during spring and summer to the previous months (Menzel et al., 2006; Wu et al., 2021)—which emphasizes the cause and effect in vegetation response to soil moisture and precipitation deficits during persistent heat waves in warm-dry regions (Klesse et al., 2018). Particularly, the Mediterranean suffers from decreasing precipitation trends and rising temperatures. Cramer et al. (2018) highlighted the strong coupling of temperature increase and potential precipitation decline of up to 30% by 2080, accompanied by a 10–20% increase in heavy rainfall events (Cramer et al., 2018) and an increase in heat wave related harvest loss of 3%/year (Brás et al., 2021). In addition, Winkler et al. (2021) emphasized the strong latitudinal gradient of climate change effects on biomes and highlighted the linkages between northern greening and warming trends compared to southern precipitation variability and subsequent browning (Winkler et al., 2021).

Parallel to this, the number of extreme years with heat waves is growing, which will raise questions regarding a calculable risk of future agricultural strategies. Central Europe is particularly affected, showing a strong increase in negative vegetation anomalies. These trends will intensify in the future and a reversal of the greening trend in the wake of prolonged droughts could lead to massive surface atmosphere interactions and regional climate amplifications. Further trend analyses of climate variables are needed to enhance the knowledge of regional environmental feedbacks. Monitoring surface transformations over long temporal periods is mostly limited by the scale and detailed ground-based measurements can be carried out only locally, which produces high spatial resolution datasets but lacks the comparison of global environmental response to global feedbacks (Cortés et al., 2021).

## Conclusion

Spectral greening shows increasing trends across most parts of Central and Northern Europe. In general, these have been linked to increasing atmospheric carbon content and rising temperatures—particularly in the northern latitudes. Simultaneously, climate variability is increasing rapidly, with higher frequencies of prolonged drought spells and severe flooding events caused by extreme weather events. Under climate change predictions, the temperature will further rise in most parts of Europe, which in turn accelerates moisture transport, soil moisture content, and eventually a longer growing season in the northern parts of Europe. On the other hand, the Mediterranean and large parts of Eastern and Southeastern Europe experience severe dry-up processes and strongly negative soil moisture trends, which impact natural vegetation and require massive irrigation measures to cope with rising surface temperatures, particularly in summer. These trends, however, do not remain restricted to the southern parts of Europe but affect Central and Eastern Europe and particularly Germany, Poland, and the central-eastern countries towards Ukraine. Landcover change caused by vegetation decline is already present in large parts of Russia and Ukraine and will further strengthen during persistent hot drought events in summer.

## Data Availability

All data underlying the results of this article are publicly available on the Internet.

## Code availability

All code underlying the analysis can be accessed at 10.5281/zenodo.7401219 (Kempf, 2022).
